# Relation between *Helicobacter pylori* infection and menopausal symptoms in diabetic women

**DOI:** 10.1186/s12905-026-04400-7

**Published:** 2026-04-21

**Authors:** Nessren Mohamed Bahaa El Deen Mohamed, Noura Ali Ghazy Zidan, Sara Mohammed Foaud Hussein, Eman Hussein Soliman Altaweel, Neamat Abdelmageed Abdelmageed, Fatma Saffeyeldin Mohamed, Sally Abdelaziz Ahmed, Marwa Abdelaziz Abo Saree Yassien, Doaa Mohamed Salama Abdelmaqsoud, Sabah M. Alkhawagah, Shymaa M. Elattar, Marwa M. Hassan, Moshira Ali Ibrahim Mohamed

**Affiliations:** 1 Hepatogastroenterology and Infectious Diseases Department, Al-Azhar University, Cairo, Egypt; 2 Department of Medicine, College of Medicine, Taif University, Taif, Saudi Arabia; 3 Endocrinology and Metabolism Department, Al-Azhar University, Cairo, Egypt; 4 Medical Microbiology and Immunology Department, Al-Azhar University, Cairo, Egypt; 5 Clinical Pathology Department, Al-Azhar University, Cairo, Egypt; 6 Internal Medicine Department, Al-Azhar University, Cairo, Egypt

**Keywords:** Diabetes mellitus, *Helicobacter pylori*, Menopausal symptoms

## Abstract

**Background and aim:**

Menopausal symptoms have marked negative impact on women’s physical and mental well-being and consumes substantial amount of health expenses. However, the relation between *H. pylori* infection and severity of menopausal symptoms is a scarcely discussed issue. The present cross-sectional study aims to explore the relation between *H. pylori* infection and severity of menopausal symptoms in diabetic women.

**Subjects and methods:**

The study included 150 postmenopausal diabetic women and 150 non-diabetic counterparts. *H. pylori* infection was assessed using a monoclonal enzyme immunoassay–based stool antigen test. Menopausal symptoms were evaluated using the Menopause Rating Scale (MRS).

**Results:**

The present study included consecutive 150 diabetic and 150 non-diabetic postmenopausal women. Diabetic women had significantly higher frequency of severe menopausal symptoms (31.3% versus 14.0%, *p*<0.001) and significantly higher frequency of positive *H. pylori* infection (56.0% versus 40.7%, *p* = 0.011). Multivariate binary logistic regression analysis identified significant association between BMI [OR (95% CI): 1.09 (1.01–1.18), *p* = 0.022], hypertension [OR (95% CI): 3.97 (1.66–9.49), *p* = 0.002], HbA1c % [OR (95% CI): 1.18 (1.02–1.36), *p* = 0.029] and positive *H. pylori* infection [OR (95% CI): 4.09 (1.52-11.0), *p* = 0.005] and severe menopausal symptoms.

**Conclusions:**

Postmenopausal diabetic women had higher frequency of *H. pylori* infection which may be related to more severe menopausal symptoms in this population.

## Introduction

 Type 2 diabetes mellitus (T2DM) is a major chronic disease with substantial economic and healthcare consequences worldwide [1]. Although slightly more men than women are affected, women often present with a higher burden of risk factors at diagnosis, including obesity, hypertension, and a history of gestational diabetes, and they are also at greater risk of cardiovascular complications and mortality [2].

The impact of T2DM on women spans all phases of the reproductive life cycle. During the childbearing years, diabetic women are at increased risk of menstrual disorders, polycystic ovary syndrome, and infertility [3]. In the postmenopausal period, T2DM is associated with several adverse outcomes, including heightened cardiovascular risk [4], osteoporosis [5], and gut microbiota dysbiosis [6].

Menopausal transition generally occurs between 45 and 56 years of age. During this period, 50–75% of women experience vasomotor symptoms (such as hot flashes and night sweats), while over 50% develop genitourinary symptoms, which may persist for many years after menopause [7]. These symptoms have a considerable negative impact on women’s physical and mental well-being and contribute substantially to healthcare utilization [8]. Notably, diabetic women appear to experience more severe menopausal symptoms compared to non-diabetic women [9].

*Helicobacter pylori* infection affects over 40% of adults worldwide [10], with a slight male predominance [11]. In premenopausal women, *H. pylori* infection has been associated with reproductive complications, including infertility, premature delivery, abortion, hyperemesis gravidarum, preeclampsia, and gestational diabetes [12, 13]. In postmenopausal women, *H. pylori* infection has been linked to osteoporosis [14]. However, the relationship between *H. pylori* infection and menopausal symptom severity remains largely unexplored. While T2DM has been associated with a higher prevalence of *H. pylori* infection in the general population [15], this association has not been adequately investigated in postmenopausal women. The present cross-sectional study aimed to explore the relation between *H. pylori* infection and severity of menopausal symptoms in diabetic women.

## Subjects and methods

The present cross-sectional comparative study was conducted at the Faculty of Medicine, Al-Azhar University, Cairo, Egypt. The institutional review board provided ethical approval and informed written consent was obtained from all participants in accordance with the regulations of the Helsinki Declaration on clinical research involving human subjects.

The study included consecutive 150 postmenopausal diabetic women and 150 non-diabetic counterparts recruited from the outpatient clinics of Al-Azhar University Hospitals. The sample size was calculated to detect a clinically significant difference between groups, taking several factors into account. First, there are limited published data on *H. pylori* prevalence specifically in postmenopausal diabetic women, either in Egypt or worldwide. Second, previous studies have reported a wide range of prevalence of *H. pylori* among diabetic and non-diabetic patients [16], and third, *H. pylori* prevalence increases with age [17], further supporting the need for adequate sample size.

Based on these considerations, assuming a baseline prevalence of 50% in the control group, 124 participants per group would be sufficient to detect a 20% absolute difference in prevalence between diabetic and non-diabetic women with 90% power at a 5% significance level (two-tailed test). To account for potential dropouts or incomplete data, an additional 20% was added, resulting in a final sample size of 150 participants per group (300 total). Sample size calculations were performed using G*Power version 3.1.9.7 (Heinrich-Heine-Universität Düsseldorf, Germany) [18].

Women were included in the study if they are in their postmenopausal stage defined by absence of menstrual bleeding during the last 12 months [19]. T2DM was diagnosed on the basis of the criteria suggested by the American Diabetic Association [20]. Participants were excluded if they were under hormonal replacement therapy, received *H. pylori* eradication therapy or other systemic antibiotics within the previous three months. Patients were also excluded if they have malignant tumors immunocompromised conditions, cognitive impairment or conditions commonly associated with cognitive impairment e.g. depression and anxiety.

All participants subjected to careful history taking, thorough clinical examination and standard laboratory work up including complete blood picture, glycemic profile, lipid profile, liver and renal functions. *H. pylori* infection was assessed using a monoclonal enzyme immunoassay–based stool antigen test (HpSA). This non-invasive diagnostic method has been shown to have high diagnostic accuracy, with reported sensitivity and specificity generally exceeding 90% for the detection of active *H. pylori* infection [21]. The test was performed and interpreted in accordance with the manufacturer’s instructions, and results were recorded as positive or negative based on predefined cutoff values. The socioeconomic status of included patients was assessed using a validated scale [22].

Menopausal symptoms were evaluated using the Menopause Rating Scale (MRS), a validated self-administered questionnaire comprising 11 items across three domains: somatic, psychological, and urogenital. Each item is scored on a 5-point Likert scale ranging from 0 (no symptoms) to 4 (very severe symptoms). The total MRS score is calculated by summing all item scores, yielding a possible range from 0 to 44, with higher scores indicating greater symptom severity. Based on the total score, menopausal symptom severity is commonly categorized as none or minimal (0–4), mild (5–8), moderate (9–16), and severe (≥ 17). The MRS has demonstrated good reliability, construct validity, and cross-cultural applicability and is widely used to assess menopausal symptom burden and treatment outcomes in both clinical practice and research settings [23].

Statistical analysis was performed using the Statistical Package for the Social Sciences (SPSS), version 27.0 (IBM Corp., Armonk, NY, USA). Continuous variables were tested for normality and presented as mean ± standard deviation. Comparisons between two independent groups were conducted using the independent samples *t*-test, while comparisons among more than two groups were performed using one-way analysis of variance (ANOVA), followed by appropriate post hoc tests when indicated. Categorical variables were expressed as frequencies and percentages and compared using the chi-square test or Fisher’s exact test when expected cell counts were less than five. Binary logistic regression analysis was used to identify independent predictors of the outcome of interest, with results presented as odds ratios (ORs) and 95% confidence intervals (CIs). A two-tailed *p* value of < 0.05 was considered statistically significant.

## Results

The present study included 150 diabetic and 150 non-diabetic postmenopausal women. Comparison between the studied groups regarding the clinical and laboratory data is shown in Table 1. Diabetic women had significantly higher BMI (34.5 ± 6.1 Kg/m^2^ versus 28.8 ± 5.9, *p*<0.001), significantly higher frequency of obesity (82.0% versus 42.7%, <0.001), higher cholesterol levels (208.2 ± 47.7 mg/dL versus 175.8 ± 34.9, *p*<0.001), higher HbA1c levels (8.0 ± 2.9% versus 5.3 ± 0.3, *p*<0.001). In addition, they had significantly higher MRS (14.4 ± 11.1 versus 10.0 ± 8.6, *p*<0.001), significantly higher frequency of severe menopausal symptoms (31.3% versus 14.0%, *p*<0.001) and significantly higher frequency of positive *H. pylori* infection (56.0% versus 40.7%, *p* = 0.011).

**Table 1:** Clinical and laboratory findings in the studies groups

	Diabetics *N* = 150	Non-diabetics *N* = 150	*p* value
Age (years) mean ± SD	57.0 ± 5.6	55.0 ± 4.6	0.2
Age at menopause (years) mean ± SD	49.4 ± 3.3	48.0 ± 5.4	0.11
Time since menopause (years) mean ± SD	7.6 ± 4.8	7.0 ± 5.9	0.3
Socioeconomic status *n* (%)			
Low	75 (50.0)	69 (46.0)	0.71
Middle	56 (37.3)	63 (42.0)	
High	19 (12.7)	18 (12.0)	
BMI (Kg/m^2^) mean ± SD	34.5 ± 6.1	28.8 ± 5.9	<0.001
Diabetes duration (years) mean ± SD	8.7 ± 3.4	-	-
Associated morbidities *n* (%)			
Obesity	123 (82.0)	64 (42.7)	<0.001
IHD	19 (12.7)	9 (6.0)	0.07
Hypertension	76 (50.7)	65 (43.3)	0.25
CLD	2 (1.3)	2 (1.3)	1.0
Laboratory findings mean ± SD			
Hb gm/dL	11.5 ± 1.3	11.7 ± 2.0	0.43
TLC ×10^3^/mL	7.4 ± 1.7	7.1 ± 1.4	0.17
Platelets ×10^3^/mL	257.5 ± 54.7	273.2 ± 47.9	0.18
Cholesterol mg/dL	208.2 ± 47.7	175.8 ± 34.9	<0.001
Triglycerides mg/dL	145.5 ± 61.6	127.4 ± 36.9	0.002
LDL mg/dL	116.3 ± 40.4	92.8 ± 26.9	<0.001
HDL mg/dL	47.6 ± 8.9	54.2 ± 14.2	<0.001
FBS mg/dL	163.0 ± 59.8	88.4 ± 7.4	<0.001
PPBS mg/dL	212.7 ± 76.1	125.2 ± 7.2	<0.001
HbA1c %	8.0 ± 2.9	5.3 ± 0.3	<0.001
Albumin gm/dL	4.2 ± 0.4	4.3 ± 0.4	0.11
Creatinine mg/dL	0.8 ± 0.1	0.8 ± 0.3	0.69
Bilirubin mg/dL	0.9 ± 0.3	0.9 ± 0.2	0.57
MRS mean ± SD	14.4 ± 11.1	10.0 ± 8.6	<0.001
Severity of menopausal symptoms *n* (%)			
None/minimal	31 (20.7)	34 (22.7)	<0.001
Mild	28 (18.7)	60 (40.0)	
Moderate	44 (29.3)	35 (23.3)	
Severe	47 (31.3)	21 (14.0)	
*H. pylori* positive *n* (%)	84 (56.0)	61 (40.7)	0.011

Continuous data were compared using t test, one way ANOVA while categorical data were compared using Fisher’s exact test or chi-square test

Abbreviations: *BMI* Body mass index, *CLD* Chronic lung disease, *FBS* Fasting blood sugar, *Hb* Hemoglobin, *HbA1c* Hemoglobin A1c, *HDL* High-density lipoprotein, *IHD* Ischemic heart disease, *LDL* Low-density lipoprotein, *MRS* Menopause rating scale, *PPBS* Postprandial blood sugar, *TLC* Total leucocytic count

**Fig. 1:**
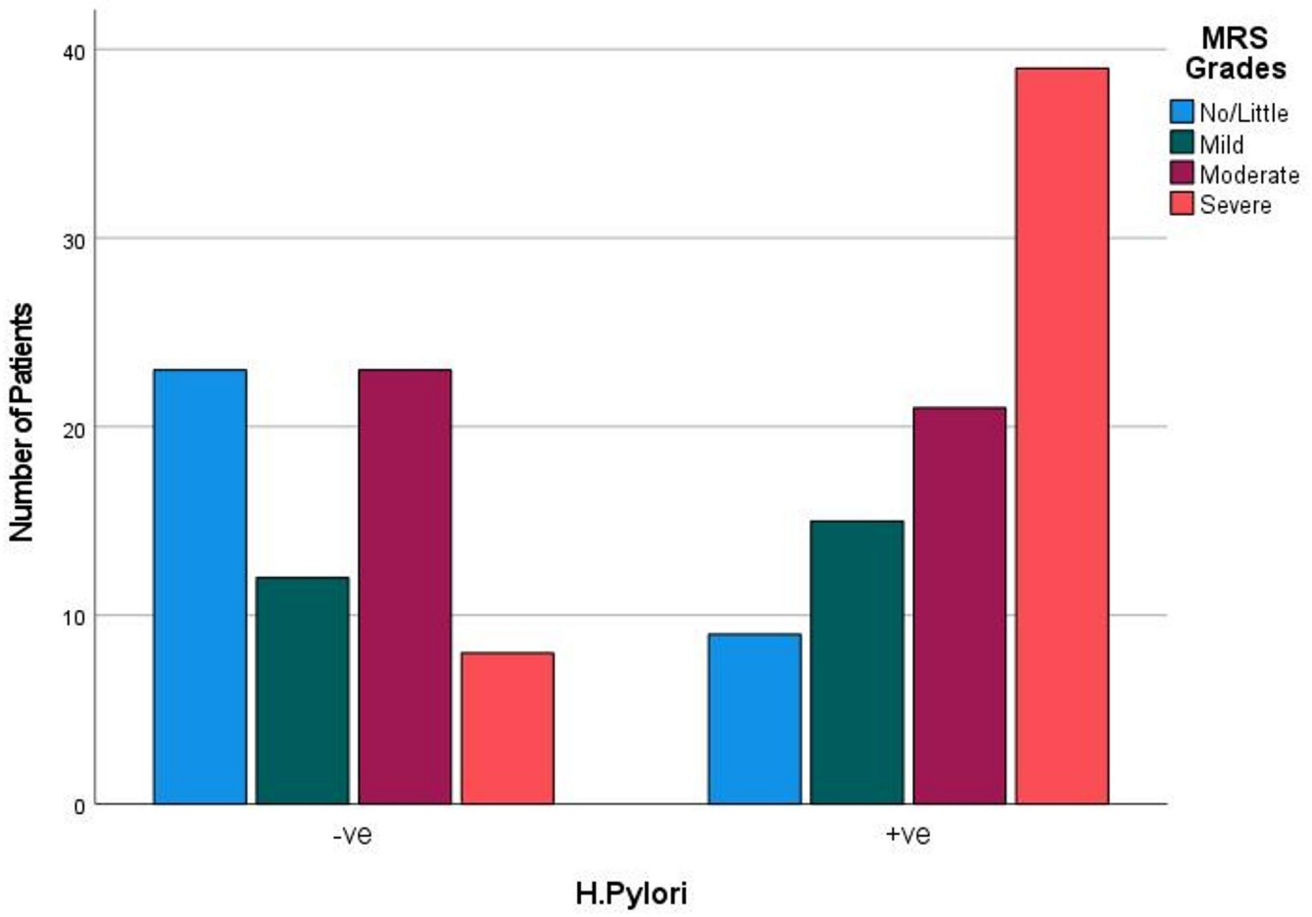
Relation between *H. pylori* infection and severity of menopausal symptoms

Comparison between diabetic patients with positive *H. pylori* infection and patients without revealed that the former subgroup comprised significantly higher frequency of obesity (88.1% versus 74.2%, *p* = 0.034), higher cholesterol levels (225.4 ± 44.2 mg/dL versus 186.3 ± 43.1, *p*<0.001), significantly higher HbA1c levels (8.7 ± 3.0% versus 7.0 ± 2.4, *p*<0.001) and significantly higher MRS (17.9 ± 11.7 versus 9.7 ± 8.6, *p*<0.001). Moreover, they included significantly higher frequency of patients with severe menopausal symptoms (46.4% versus 12.1%, *p*<0.001) (Table 2; Fig. 1).

**Table 2:** Relation between *H. pylori* infection and clinical and laboratory findings in diabetic patients

	H. pylori positive *N* = 84	H. pylori negative *N* = 66	*p* value
Age (years) mean ± SD	56.7 ± 5.1	57.3 ± 6.2	0.5
Age at menopause (years) mean ± SD	49.3 ± 2.8	49.5 ± 3.8	0.7
Time since menopause (years) mean ± SD	7.4 ± 4.6	7.8 ± 5.0	0.59
Socioeconomic status *n* (%)			
Low	40 (47.6)	35 (53.0)	0.73
Middle	32 (38.1)	24 (36.4)	
High	12 (14.3)	3 (4.6)	
BMI (Kg/m^2^) mean ± SD	35.3 ± 5.9	33.4 ± 6.2	0.052
Diabetes duration (years) mean ± SD	8.7 ± 3.4	8.3 ± 3.2	0.44
Associated morbidities *n* (%)			
Obesity	74 (88.1)	49 (74.2)	0.034
IHD	8 (9.5)	11 (16.7)	0.22
Hypertension	43 (51.2)	33 (50.0)	0.89
CLD	-	2 (3.0)	0.19
Laboratory findings mean ± SD			
Hb gm/dL	11.6 ± 1.3	11.5 ± 1.2	0.82
TLC ×10^3^/mL	7.5 ± 1.7	7.3 ± 1.7	0.37
Platelets ×10^3^/mL	251.8 ± 51.1	264.8 ± 58.6	0.15
Cholesterol mg/dL	225.4 ± 44.2	186.3 ± 43.1	<0.001
Triglycerides mg/dL	164.8 ± 62.4	121.0 ± 51.5	<0.001
LDL mg/dL	128.5 ± 41.9	100.8 ± 32.6	<0.001
HDL mg/dL	46.1 ± 8.9	49.5 ± 8.7	0.021
FBS mg/dL	171.0 ± 64.3	152.9 ± 52.4	0.059
PPBS mg/dL	220.3 ± 82.9	203.1 ± 65.8	0.16
HbA1c %	8.7 ± 3.0	7.0 ± 2.4	<0.001
Albumin gm/dL	4.1 ± 0.4	4.2 ± 0.3	0.86
Creatinine mg/dL	0.9 ± 0.2	0.8 ± 0.3	0.23
Bilirubin mg/dL	0.9 ± 0.3	0.9 ± 0.2	0.85
MRS mean ± SD	17.9 ± 11.7	9.7 ± 8.6	<0.001
Severity of menopausal symptoms *n* (%)			
None/minimal	9 (10.7)	23 (34.9)	<0.001
Mild	15 (17.9)	12 (18.2)	
Moderate	21 (25.0)	23 (34.9)	
Severe	39 (46.4)	8 (12.1)	

Continuous data were compared using t test, one way ANOVA while categorical data were compared using Fisher’s exact test or chi-square test

Diabetic patients were classified into three subgroups according to MRS: none/minimal-to-mild (*n* = 59), moderate (*n* = 44) and severe (*n* = 47). Patients with severe menopausal symptoms were found to have significantly higher BMI (36.8 ± 6.4 Kg/m^2^) in comparison to those with none/minimal-to-mild and moderate symptoms (33.9 ± 5.5 and 32.8 ± 5.9 Kg/m^2^ respectively, *p* = 0.004). In addition, they comprised significantly higher frequency of hypertensive patients (68.1% versus 42.4% and 43.2% respectively, *p* = 0.016). Likewise, they had significantly higher cholesterol levels (229.7 ± 48.1 mg/dL versus 201.4 ± 42.5 and 194.2 ± 47.1, *p*<0.001) and HbA1c levels (9.2 ± 2.7% versus 7.4 ± 2.4 and 7.3 ± 3.3) when compared with the other two subgroups. Moreover, it was found that patients with severe menopausal symptoms had significantly higher frequency of positive *H. pylori* infection as compared to the other two subgroups (83.0% versus 40.7% and 47.7%, *p*<0.001) (Table 3).

**Table 3:** Relation between severity of menopausal symptoms and clinical and laboratory findings in diabetic patients

	None/minimal-to-mild *N* = 59	Moderate *N* = 44	Severe *N* = 47	*p* value
Age (years) mean ± SD	58.2 ± 6.2	56.4 ± 4.5	55.9 ± 5.6	0.081
Age at menopause (years) mean ± SD	49.9 ± 3.3	49.0 ± 3.0	49.1 ± 3.5	0.3
Time since menopause (years) mean ± SD	8.4 ± 4.4	7.5 ± 4.9	6.8 ± 5.1	0.26
Socioeconomic status *n* (%)				
Low	29 (49.2)	21 (47.7)	25 (53.2)	0.98
Middle	22 (37.3)	17 (38.6)	17 (36.2)	
High	8 (13.5)	6 (13.6)	5 (10.6)	
BMI (Kg/m^2^) mean ± SD	33.9 ± 5.5*	32.8 ± 5.9*	36.8 ± 6.4	0.004
Diabetes duration (years) mean ± SD	8.5 ± 3.5	8.8 ± 3.2	8.3 ± 3.3	0.73
Associated morbidities *n* (%)				
Obesity	47 (79.7)	34 (77.3)	42 (89.4)	0.27
IHD	6 (10.2)	9 (20.5)	4 (8.5)	0.18
Hypertension	25 (42.4)	19 (43.2)	32 (68.1)	0.016
CLD	-	1 (2.3)	1 (2.1)	0.52
Laboratory findings mean ± SD				
Hb gm/dL	11.3 ± 1.1	11.9 ± 1.3	11.4 ± 1.4	0.44
TLC ×10^3^/mL	7.6 ± 1.9	7.3 ± 1.7	7.3 ± 1.5	0.53
Platelets ×10^3^/mL	267.4 ± 64.3	243.5 ± 46.7	258.2 ± 46.1	0.088
Cholesterol mg/dL	201.4 ± 42.5*	194.2 ± 47.1*	229.7 ± 48.1	<0.001
Triglycerides mg/dL	133.3 ± 57.8*	137.9 ± 62.2*	168.0 ± 60.8	0.009
LDL mg/dL	110.8 ± 33.5	111.9 ± 46.2	127.4 ± 41.2	0.074
HDL mg/dL	49.8 ± 8.4*	48.7 ± 8.5*	43.9 ± 8.9	0.002
FBS mg/dL	161.5 ± 53.2	167.4 ± 72.6	160.8 ± 55.4	0.85
PPBS mg/dL	227.5 ± 75.6	198.1 ± 72.8	208.0 ± 78.2	0.14
HbA1c %	7.4 ± 2.4*	7.3 ± 3.3*	9.2 ± 2.7	0.002
Albumin gm/dL	4.1 ± 0.3	4.2 ± 0.4	4.1 ± 0.4	0.4
Creatinine mg/dL	0.8 ± 0.2	0.8 ± 0.2	0.9 ± 0.3	0.074
Bilirubin mg/dL	0.9 ± 0.1	1.0 ± 0.3	0.9 ± 0.2	0.67
*H. pylori* positive *n* (%)	24 (40.7)	21 (47.7)	39 (83.0)	<0.001

Continuous data were compared using t test, one way ANOVA while categorical data were compared using Fisher’s exact test or chi-square test

* Significant results versus severe subgroup

Multivariate binary logistic regression analysis identified significant association between BMI [OR (95% CI): 1.09 (1.01–1.18), *p* = 0.022], hypertension [OR (95% CI): 3.97 (1.66–9.49), *p* = 0.002], HbA1c % [OR (95% CI): 1.18 (1.02–1.36), *p* = 0.029] and positive *H. pylori* infection [OR (95% CI): 4.09 (1.52-11.0), *p* = 0.005] and severe menopausal symptoms (Table 4).

**Table 4:** Predictors of severe menopausal symptoms in diabetic patients

	Univariate analysis			Multivariate analysis		
	OR	95% CI	*p* value	OR	95% CI	*p* value
BMI	1.11	1.04–1.19	0.001	1.09	1.01–1.18	0.022
Hypertension	3.11	1.49–6.53	0.003	3.97	1.66–9.49	0.002
Cholesterol	1.02	1.01–1.03	<0.001	1.01	0.99–1.02	0.23
HbA1c %	1.2	1.1–1.4	<0.001	1.18	1.02–1.36	0.029
*H. pylori* positive	6.0	2.5–14.1	<0.001	4.09	1.52-11.0	0.005

Binary logistic regression was used to determine associations with severe menopausal symptoms

## Discussion

The present study aimed to shed light on a rarely discussed issue in the particularly vulnerable group of postmenopausal diabetic women. The study didn’t only assess the prevalence of *H. pylori* infection in those patients but also investigation the relation between this infection and severity of postmenopausal symptoms. The study found that postmenopausal diabetic women had significantly higher frequency of severe menopausal symptoms as compared to non-diabetic counterparts. In line with this finding, the recent study of Armeni et al. [9] concluded that the severity of menopausal symptoms is associated with T2DM in peri-/postmenopausal women. In contrast, Rouen et al. [24] found no association between T2DM and severity of menopausal symptoms.

Moreover, the study found that diabetic women had significantly higher frequency of positive *H. pylori* infection when compared with non-diabetics. This in harmony with the conclusions of one meta-analysis including 41 studies involving 9559 individuals. The authors found a positive association between *H. pylori* infection and diabetes mellitus [25].

Comparing diabetic patients with and without *H. pylori* infection showed that a significant relation between positive *H. pylori* and obesity, dyslipidemia and poor glycemic control. In line with these results, Baradaran et al. [26] reported a positive correlation between *H. pylori* infection and the prevalence of obesity in their 25,519-patient meta-analysis. Also, the recent meta-analysis of Gaonkar et al. [27] found that *H. pylori* infection is associated with significant dyslipidemia. In addition, multiple studies found a link between *H. pylori* infection and poor glycemic control [28, 29].

Regarding the relationship between the severity of menopausal symptoms and *H. pylori* infection, the present study identified significant association between *Helicobacter pylori* infection and severe menopausal symptoms. To the best of our knowledge, this represents a novel finding.

Several mechanisms may explain this association. First, *H. pylori* infection has been linked to chronic long-term inflammation, leading to the sustained release of pro-inflammatory and immune-disrupting mediators [30]. The association between chronic systemic inflammation and increased severity of menopausal symptoms has been documented in a recent study [31]. Second, *H. pylori* infection has been associated with nutritional deficiencies, particularly vitamin B12 deficiency [32], which has been linked to several menopausal symptoms, including fatigue, cognitive impairment, and mood disturbances [33]. Moreover, *H. pylori* infection is believed to exert neuropsychiatric effects through modulation of the gut–brain axis [34], potentially contributing to the development of mood changes and other psychological symptoms commonly observed during menopause [35].

In conclusion, the present study found that postmenopausal diabetic women had higher frequency of *H. pylori* infection which may be related to more severe menopausal symptoms in this population suggesting that evaluation of *H. pylori* infection in those patients may be clinically useful particularly in patients not responding to standard treatment.

## Limitations and recommendations

Conclusions of the present study should be interpreted with caution due to the cross-sectional design of the study, which precludes establishing causal relationships. Furthermore, certain potential confounders, such as medication use, were not systematically assessed because of substantial heterogeneity and the high prevalence of polypharmacy within the study population. Additionally, as this was a single-center study that recruited participants from an outpatient clinic setting, the generalizability of the findings may be limited. Further population-based studies multicentric study are recommended to confirm the study findings. Longitudinal and mechanistic studies are also warranted to elucidate the pathophysiological mechanisms underlying the association between *H. pylori* infection and the severity of menopausal symptoms in postmenopausal women with diabetes. Targeting *H. pylori* infection in women with severe menopausal symptoms requires well-designed randomized studies to confirm the benefit of such an approach.

## Data Availability

Data will be available from the corresponding author upon reasonable request.

## References

[1] Tinajero MG, Malik VS. An Update on the Epidemiology of Type 2 Diabetes: a Global Perspective. Endocrinol Metab Clin North Am. 2021;50(3):337–355. 10.1016/j.ecl.2021.05.013. PMID: 34399949. 34399949 10.1016/j.ecl.2021.05.013

[2] Kautzky-Willer A, Leutner M, Harreiter J. Sex differences in type 2 diabetes. Diabetologia. 2023;66(6):986-1002. 10.1007/s00125-023-05891-x. Epub 2023 Mar 10. Erratum in: Diabetologia. 2023;66(6):1165. https://doi.org/10.1007/s00125-023-05913-8. PMID: 36897358; PMCID: PMC10163139. 10.1007/s00125-023-05891-x PMC10163139 36897358

[3] Zaimi M, Michalopoulou O, Stefanaki K, Kazakou P, Vasileiou V, Psaltopoulou T, Karagiannakis DS, Paschou SA. Gonadal dysfunction in women with diabetes mellitus. Endocrine. 2024;85(2):461–72. 10.1007/s12020-024-03729-z. Epub 2024 Feb 14. PMID: 38353886; PMCID: PMC11291547. 38353886 10.1007/s12020-024-03729-z PMC11291547

[4] Rodriguez de Morales YA, Abramson BL. Cardiovascular and physiological risk factors in women at mid-life and beyond. Can J Physiol Pharmacol. 2024;102(8):442–51. 10.1139/cjpp-2023-0468. Epub 2024 May 13. PMID: 38739947. 38739947 10.1139/cjpp-2023-0468

[5] Li J, Li Z, Li S, Lu Y, Li Y, Rai P. Correlation of metabolic markers and OPG gene mutations with bone mass abnormalities in postmenopausal women. J Orthop Surg Res. 2024;19(1):706. 10.1186/s13018-024-05162-4. PMID: 39487469; PMCID: PMC11529261. 39487469 10.1186/s13018-024-05162-4 PMC11529261

[6] Singh V, Park YJ, Lee G, Unno T, Shin JH. Dietary regulations for microbiota dysbiosis among post-menopausal women with type 2 diabetes. Crit Rev Food Sci Nutr. 2023;63(29):9961–76. Epub 2022 May 30. PMID: 35635755. 35635755 10.1080/10408398.2022.2076651

[7] Crandall CJ, Mehta JM, Manson JE. Management of menopausal symptoms: a review. JAMA. 2023;329(5):405–420. 10.1001/jama.2022.24140. PMID: 36749328. 36749328 10.1001/jama.2022.24140

[8] Patel B, Dhillo S. Menopause review: Emerging treatments for menopausal symptoms. Best Pract Res Clin Obstet Gynaecol. 2022;81:134–44. 10.1016/j.bpobgyn.2021.10.010. Epub 2021 Nov 17. PMID: 34965909. 34965909 10.1016/j.bpobgyn.2021.10.010

[9] Armeni E, Kopanos S, Verykouki E, Augoulea A, Paschou SA, Rizos D, Kaparos G, Eleftheriadis M, Haidich AB, Goulis DG, Vlahos N, Lambrinoudaki I. The severity of menopausal symptoms is associated with diabetes, and cardiometabolic risk factors in middle-aged women. Minerva Endocrinol (Torino). 2023. 10.23736/S2724-6507.23.03905-2. Epub ahead of print. PMID: 37671810. 10.23736/S2724-6507.23.03905-2 37671810

[10] Chen YC, Malfertheiner P, Yu HT, Kuo CL, Chang YY, Meng FT, Wu YX, Hsiao JL, Chen MJ, Lin KP, Wu CY, Lin JT, O’Morain C, Megraud F, Lee WC, El-Omar EM, Wu MS, Liou JM. Global Prevalence of *Helicobacter pylori* Infection and Incidence of Gastric Cancer Between 1980 and 2022. Gastroenterology. 2024;166(4):605–19. 10.1053/j.gastro.2023.12.022. Epub 2024 Jan 2. PMID: 38176660. 38176660 10.1053/j.gastro.2023.12.022

[11] Ibrahim A, Morais S, Ferro A, Lunet N, Peleteiro B. Sex-differences in the prevalence of Helicobacter pylori infection in pediatric and adult populations: Systematic review and meta-analysis of 244 studies. Dig Liver Dis. 2017;49(7):742–9. . 10.1016/j.dld.2017.03.019 Epub 2017 Apr 4. PMID: 28495503. 28495503 10.1016/j.dld.2017.03.019

[12] Zhou B, Wang F. Research progress in relation of *Helicobacter pylori* infection with pregnancy-related diseases and adverse pregnancy outcomes. Zhong Nan Da Xue Xue Bao Yi Xue Ban. 2020;45(3):338–344. English, Chinese. 10.11817/j.issn.1672-7347.2020.190032. PMID: 32386028. 10.11817/j.issn.1672-7347.2020.190032 32386028

[13] Kohnepoushi P, Mansouri R, Moghaddam AB, Soheili M, Kohan HG, Moradi Y. The association between the *Helicobacter pylori* infection and the occurrence of gestational diabetes: a systematic review and meta-analysis. J Health Popul Nutr. 2024;43(1):136. 10.1186/s41043-024-00630-3. PMID: 39217374; PMCID: PMC11366142. 39217374 10.1186/s41043-024-00630-3 PMC11366142

[14] Kim TJ, Lee H, Min YW, Min BH, Lee JH, Rhee PL, Kim JJ. Cohort study of *Helicobacter pylori* infection and the risk of incident osteoporosis in women. J Gastroenterol Hepatol. 2021;36(3):657–63. 10.1111/jgh.15181. Epub 2020 Jul 23. PMID: 32656854. 32656854 10.1111/jgh.15181

[15] Sahoo OS, Mitra R, Bhattacharjee A, Kar S, Mukherjee O. Is Diabetes Mellitus a Predisposing Factor for *Helicobacter pylori* Infections? Curr Diab Rep. 2023;23(8):195–205. 10.1007/s11892-023-01511-5. Epub 2023 May 22. PMID: 37213058. 37213058 10.1007/s11892-023-01511-5

[16] Mansori K, Dehghanbanadaki H, Naderpour S, Rashti R, Moghaddam AB, Moradi Y. A systematic review and meta-analysis of the prevalence of Helicobacter pylori in patients with diabetes. Diabetes Metab Syndr. 2020;14(4):601–607. 10.1016/j.dsx.2020.05.009 Epub 2020 May 11. PMID: 32417710. 32417710 10.1016/j.dsx.2020.05.009

[17] Skokowski J, Vashist Y, Girnyi S, Cwalinski T, Mocarski P, Antropoli C, Brillantino A, Boccardi V, Goyal A, Ciarleglio FA, Almohaimeed MA, De Luca R, Abou-Mrad A, Marano L, Oviedo RJ, Januszko-Giergielewicz B. The Aging Stomach: Clinical Implications of H. pylori Infection in Older Adults-Challenges and Strategies for Improved Management. Int J Mol Sci. 2024;25(23):12826. 10.3390/ijms252312826. PMID: 39684537; PMCID: PMC11641014. 39684537 10.3390/ijms252312826 PMC11641014

[18] Faul F, Erdfelder E, Lang AG, Buchner A. G*Power 3: a flexible statistical power analysis program for the social, behavioral, and biomedical sciences. Behav Res Methods. 2007;39(2):175 – 91. 10.3758/bf03193146. PMID: 17695343. 17695343 10.3758/bf03193146

[19] Harlow SD, Gass M, Hall JE, Lobo R, Maki P, Rebar RW, Sherman S, Sluss PM, de Villiers TJ, STRAW + 10 Collaborative Group. Executive summary of the Stages of Reproductive Aging Workshop + 10: addressing the unfinished agenda of staging reproductive aging. J Clin Endocrinol Metab. 2012;97(4):1159–68. 10.1210/jc.2011-3362. Epub 2012 Feb 16. PMID: 22344196; PMCID: PMC3319184. 22344196 10.1210/jc.2011-3362 PMC3319184

[20] American Diabetes Association Professional Practice Committee. 2. Classification and Diagnosis of Diabetes: Standards of Medical Care in Diabetes-2022. Diabetes Care. 2022;45(Suppl 1):S17-S38. 10.2337/dc22-S002. PMID: 34964875. 10.2337/dc22-S002 34964875

[21] Gisbert JP, de la Morena F, Abraira V. Accuracy of monoclonal stool antigen test for the diagnosis of *H. pylori* infection: a systematic review and meta-analysis. Am J Gastroenterol. 2006;101(8):1921-30. 10.1111/j.1572-0241.2006.00668.x. Epub 2006 Jun 16. Erratum in: Am J Gastroenterol. 2006;101(10):2445. PMID: 16780557. 10.1111/j.1572-0241.2006.00668.x 16780557

[22] El-Gilany A, El-Wehady A, El-Wasify M. Updating and validation of the socioeconomic status scale for health research in Egypt. East Mediterr Health J. 2012;18(9):962-8. 10.26719/2012.18.9.962. PMID: 23057390. 23057390 10.26719/2012.18.9.962

[23] Heinemann LA, DoMinh T, Strelow F, Gerbsch S, Schnitker J, Schneider HP. The Menopause Rating Scale (MRS) as outcome measure for hormone treatment? A validation study. Health Qual Life Outcomes. 2004;2:67. 10.1186/1477-7525-2-67. PMID: 15555079; PMCID: PMC534786. 15555079 10.1186/1477-7525-2-67 PMC534786

[24] Rouen PA, Krein SL, Reame NE. Postmenopausal Symptoms in Female Veterans with Type 2 Diabetes: Glucose Control and Symptom Severity. J Womens Health (Larchmt). 2015;24(6):496–505. 10.1089/jwh.2014.4863. Epub 2015 May 4. PMID: 25938989; PMCID: PMC4490769. 25938989 10.1089/jwh.2014.4863 PMC4490769

[25] Mansori K, Moradi Y, Naderpour S, Rashti R, Moghaddam AB, Saed L, Mohammadi H. *Helicobacter pylori* infection as a risk factor for diabetes: a meta-analysis of case-control studies. BMC Gastroenterol. 2020;20(1):77. 10.1186/s12876-020-01223-0. PMID: 32209055; PMCID: PMC7092473. 32209055 10.1186/s12876-020-01223-0 PMC7092473

[26] Baradaran A, Dehghanbanadaki H, Naderpour S, Pirkashani LM, Rajabi A, Rashti R, Riahifar S, Moradi Y. The association between *Helicobacter pylori* and obesity: a systematic review and meta-analysis of case-control studies. Clin Diabetes Endocrinol. 2021;7(1):15. 10.1186/s40842-021-00131-w. PMID: 34243821; PMCID: PMC8272347. 34243821 10.1186/s40842-021-00131-w PMC8272347

[27] Gaonkar A, Zahiruddin QS, Shabil M, Menon SV, Kaur M, Kumari M, Sudan P, Naidu KS, Thapliyal S, Uikey J, Kathuria R, Chauhan SS, Verma L, Sidhu A, Bushi G, Yusoff RBM, Mehta R, Satapathy P, Sah S. Association of *Helicobacter pylori* Infection and Risk of Dyslipidemia: A Systematic Review and Meta-Analysis. JGH Open. 2025;9(3):e70128. 10.1002/jgh3.70128. PMID: 40130085; PMCID: PMC11931453. 40130085 10.1002/jgh3.70128 PMC11931453

[28] Chen Y, Yang C, You N, Zhang J. Relationship between *Helicobacter pylori* and glycated hemoglobin: a cohort study. Front Cell Infect Microbiol. 2023;13:1196338. 10.3389/fcimb.2023.1196338. PMID: 37360526; PMCID: PMC10288807. 37360526 10.3389/fcimb.2023.1196338 PMC10288807

[29] Dai YN, Yu WL, Zhu HT, Ding JX, Yu CH, Li YM. Is *Helicobacter pylori* infection associated with glycemic control in diabetics? World J Gastroenterol. 2015;21(17):5407–16. 10.3748/wjg.v21.i17.5407. PMID: 25954115; PMCID: PMC4419082. 25954115 10.3748/wjg.v21.i17.5407 PMC4419082

[30] Maleki N, Mohammadzadeh A, Mardaneh J, Pazoki H, Nattagh-Eshtivani E. *Helicobacter pylori* infection and association with chronic diseases: A focus on cardiovascular disease, MASLD, and type 2 diabetes. Metabol Open. 2025;27:100385. 10.1016/j.metop.2025.100385. PMID: 40893911; PMCID: PMC12391821. 40893911 10.1016/j.metop.2025.100385 PMC12391821

[31] Korpe B, Kose C, Keskin HL. Systemic inflammation and menopausal symptomatology: insights from postmenopausal women. Menopause. 2024;31(11):973–8. 10.1097/GME.0000000000002433. Epub 2024 Aug 21. PMID: 39162529. 39162529 10.1097/GME.0000000000002433

[32] Lahner E, Persechino S, Annibale B. Micronutrients (Other than iron) and Helicobacter pylori infection: a systematic review. Helicobacter. 2012;17(1):1–15. 10.1111/j.1523-5378.2011.00892.x. PMID: 22221610. 22221610 10.1111/j.1523-5378.2011.00892.x

[33] Patil HS, Herlekar SS, Baiju ML. Correlation of Vitamin B12 deficiency with sensorimotor deficits in postmenopausal women: a cross-sectional, observational study. J Midlife Health. 2025;16(1):26–31. 10.4103/jmh.jmh_103_24. Epub 2025 Apr 5. PMID: 40330229; PMCID: PMC12052276. 40330229 10.4103/jmh.jmh_103_24 PMC12052276

[34] Baj J, Forma A, Flieger W, Morawska I, Michalski A, Buszewicz G, Sitarz E, Portincasa P, Garruti G, Flieger M, Teresiński G. *Helicobacter pylori* Infection and Extragastric Diseases-A Focus on the Central Nervous System. Cells. 2021;10(9):2191. 10.3390/cells10092191. PMID: 34571840; PMCID: PMC8469861. 34571840 10.3390/cells10092191 PMC8469861

[35] Gu Y, Zheng L, Kumari S, Zhang Q, Liu L, Meng G, Wu H, Bao X, Yao Z, Sun S, Wang X, Zhou M, Jia Q, Song K, Niu K. The relationship between *Helicobacter pylori* infection and depressive symptoms in the general population in China: The TCLSIH cohort study. Helicobacter. 2019;24(5):e12632. 10.1111/hel.12632. Epub 2019 Jul 22. PMID: 31332918. 31332918 10.1111/hel.12632

